# Treatment beyond progression in non-small cell lung cancer: A systematic review and meta-analysis

**DOI:** 10.3389/fonc.2022.1023894

**Published:** 2022-11-17

**Authors:** Wei-Ke Kuo, Ching-Fu Weng, Yin-Ju Lien

**Affiliations:** ^1^ Division of Respiratory Therapy and Chest Medicine, Sijhih Cathay General Hospital, Taipei, Taiwan; ^2^ Division of Pulmonary Medicine, Department of Internal Medicine, Hsinchu Cathay General Hospital, Hsinchu, Taiwan; ^3^ School of Medicine, National Tsing Hua University, Hsinchu, Taiwan; ^4^ Department of Health Promotion and Health Education, National Taiwan Normal University, Taipei, Taiwan

**Keywords:** treatment beyond progression, NSCLC, meta-analysis, systemic review, survival

## Abstract

**Objectives:**

Treatment beyond progression (TBP) is defined as treatment continuing in spite of disease progression, according to the Response Evaluation Criteria In Solid Tumors. We performed a systematic review and meta-analysis to provide evidence for the effects of TBP on lung cancer survival.

**Materials and methods:**

This study has been conducted following the PRISMA guidelines. A systematic review of PubMed, MEDLINE, Embase, and Cochrane Collaboration Central Register of Controlled Clinical Trials from the inception of each database to December 2021 was conducted. Two authors independently reviewed articles for inclusion and extract data from all the retrieved articles. Random-effects meta-analysis was performed using Comprehensive Meta-Analysis software, version 3 (Biostat, Englewood, NJ, USA). Hazard ratios (HRs) with the corresponding 95% confidence intervals (CI) were used for survival outcomes.

**Results:**

We identified five (15.6%) prospective randomized trials and twenty-seven (84.4%) retrospective observational studies of a total of 9,631 patients for the meta-analysis. 3,941 patients (40.9%) were in a TBP group and 5,690 patients (59.1%) were in a non-TBP group. There is a statistically significant advantage for patients who received TBP compared with those who did not in post progression progression-free survival (ppPFS), post progression overall survival (ppOS), and overall survival (OS) from initiation of drugs (ppPFS: HR, 0.746; 95% CI, 0.644-0.865; P<0.001; ppOS: HR, 0.689; 95% CI, 0.596-0.797; P<0.001; OS from initiation of drugs: HR, 0.515; 95% CI, 0.387-0.685; P<0.001)

**Conclusion:**

This study provides further evidence in support of TBP for NSCLC, however, these results require cautious interpretation. Large, randomized, controlled trials investigating the efficacy of TBP in lung cancer treatment are warranted.

**Systemic Review Registration:**

https://www.crd.york.ac.uk/PROSPERO/ identifier CRD42021285147

## Introduction

Lung cancer is the leading cause of cancer related mortality worldwide, with most patients having advanced disease at the time of diagnosis (1). However, much progress has been made recently in treating non-small cell lung cancer (NSCLC), the most common type of lung cancer. Tyrosine kinase inhibitors (TKIs), anti-angiogenesis agents, and immune checkpoint inhibitors have dramatically changed the landscape of NSCLC treatment (2–4).. In addition, combination therapy with different pharmaceuticals has proven highly effective due to the ability to affect multiple pathways involved in the progression of the disease (5).

Drug resistance has been the most important factor limiting the success, in terms of overall survival, of systemic anticancer therapy for advanced lung cancer. Once a cancer has developed resistance to a given chemotherapeutic agent, the usual strategy is to initiate a different therapy using non-cross resistant drugs (6). Since the Response Evaluation Criteria In Solid Tumors (RECIST) was introduced in 2000 (Version 1.0) and updated in 2009 (Version 1.1), to assess tumor response of cancer therapeutics by RECIST and to stop current anti-cancer treatment if evaluated as disease progression has been the standard of care of lung cancer management (7, 8). In a systematic review, Davies et al. described that median overall survival ranged from 4.6 months to 12.8 months from the time of second-line treatment initiation in advanced NSCLC (9). Approximately 30% of patients received third-line treatment and only 2.5 to 17.7% patients received fourth-line therapy (9). Currently, there is an unmet need to prolong the duration of each line of effective therapy.

In oncology, treatment beyond progression (TBP) is an expression that indicates the continuation of ongoing therapy after disease progression has resumed (10). TBP is therefore defined as treatment continuing in spite of disease progression, according to the Response Evaluation Criteria In Solid Tumors (RECIST). TBP is carried on while the patient tolerates the current therapy well, is clinically stable, without main organ dysfunction, and provides updated consent (11).

Randomized studies in which patients either continue or discontinue an anti-cancer agent after disease progression are essential to conclude whether TBP is effective. However, there might have substantial difference of characteristics between patients who continue a treatment and those who discontinue treatment (6). Generally, patients who are doing well are left on their medicine while those who are struggling are moved onto a new therapy. As a result, randomized controlled trials which investigate lung cancer treatment are scant. Nevertheless, there are retrospective observation studies. These studies are not designed to compare TBP or treatment discontinuation, which may result in selection bias, and have produced inconsistent results because of small sample sizes and part of subgroup analysis. However, they are still of great reference value clinically. Therefore, we conducted the present systematic review and meta-analysis to provide evidence for the effects of TBP on lung cancer survival.

## Materials and methods

This systematic review and meta-analysis have been performed following the PRISMA checklist. The study protocol was registered with the International Prospective Register of Systematic Reviews (PROSPERO) database (CRD42021285147).

### Eligibility criteria

We included clinical trials (i.e., randomized, quasi-randomized) and observational studies which investigated continuing current anti-cancer treatment beyond RECIST progression among patients with lung cancer We included patients receiving four kinds of anti-cancer treatment: targeted therapy, immunotherapy, anti-angiogenesis agents, and chemotherapy. Articles were limited to those available in full text and published in English and Chinese peer-reviewed journals.

### Search strategy

We conducted a literature search using these electronic databases: PubMed, MEDLINE, Embase, and Cochrane Collaboration Central Register of Controlled Clinical Trials from the inception of each database to December 2021. We also reviewed the bibliographies of included trials and related review articles for relevant references. The search strategy comprised the following terms (lung cancer) AND (treatment beyond progression) AND ((target therapy) OR (immunotherapy) OR (anti-angiogenesis) OR (chemotherapy)). There was no time restriction on the duration of trials.

### Study selection and data collection

Two investigators screened studies independently. All disagreement between investigators was resolved by consulting a third reviewer. Discrepancies in study inclusion were discussed among all authors until consensus was achieved. We screened the titles and abstracts identified from the electronic search and investigated full text articles of those deemed potentially relevant. All retrieved studies were required to contain at least two treatment arms, one of which was the intervention group (TBP group), and the other of which was the control group (non-TBP group). The target population consisted of NSCLC patients receiving systemic therapy, including targeted therapy, immunotherapy, anti-angiogenesis agents, and chemotherapy. Systemic therapy regarding neo-adjuvant or adjuvant settings were excluded.

### Data extraction

The two reviewers used a predetermined data extraction sheet to extract data from all the retrieved articles. We recorded study characteristics, including first author, year of publication, study design, type and details of treatment arms. We attempted to contact the corresponding author in cases where the data in the article were incompletely reported.

### Quality assessment

The two reviewers evaluated the quality of the enrolled studies independently. For non-randomized studies, we used the Risk of Bias Assessment Tool for Non-randomized Studies (RoBANS) which consists of six domains; these include selection of participants, confounding variables, measurement of exposure, blinding of outcome assessment, incomplete outcome data, and selective outcome reporting (12). We used the Revised Cochrane risk-of-bias tool for randomized trials (RoB 2). It contains five domains, including the randomization process, intended intervention, missing outcome data, measurement of outcomes, and selection of reported results. Based on the RoB 2, we evaluated methodological quality as falling into three categories: low risk of bias, some concerns, and high risk of bias.

### Outcome measures

The outcomes of interest were post progression progression-free survival (ppPFS), post progression overall survival (ppOS), and overall survival (OS) from initiation of drugs. ppPFS was defined as the time starting from the first point of disease progression following use of intervention drugs until the second progression or death; ppOS was defined as the period from the date of first disease progression (PD) after use of intervention drugs to the date of death due to any cause; OS from initiation of drugs was defined as the period from the date treatment with the intervention drugs began to the date of death due to any cause.

*A priori* subgroup analysis for the outcomes of interest were planned based on classification of the TBP intervention drugs (classified as “Epidermal Growth Factor Receptor (EGFR) TKIs”, “Anaplastic lymphoma kinase (ALK) TKIs”, “immunotherapy”, and “anti-angiogenesis agents”), treatment of the non-TBP group (classified as “Other” and “Other and None”), whether add-on therapy was allowed in the TBP group (classified as “With add-on” and “Without add-on”), and region (classified as “America”, “Asia”, “Europe”, and “Worldwide”). “Other” indicates patients who switched from TBP to other anticancer treatments such as chemotherapy and immunotherapy, and “Other and None” indicates patients who switched to other anticancer treatments plus those who received no further anticancer treatment. “Add-on” refers to a treatment strategy of continuing TBP but adding chemotherapy or radiotherapy to that.

### Analysis

Meta-analysis was performed using Comprehensive Meta-Analysis (CMA) software, version 3 (Biostat, Englewood, NJ, USA). Hazard ratios (HRs) with the corresponding 95% confidence intervals (CI) were used for survival outcomes. Because of the clinical heterogeneity inherent in the data, we employed a random effects model to pool individual HRs. We used forest plots to graphically display the effect size in each group and the pooled estimates. Between-study heterogeneity was assessed using *I* ^2^ tests; values greater than 50% were considered significant heterogeneity. We conducted sensitivity analysis to assess the impact of each study on the pooled estimate by removing each study one at a time and recalculating the pooled HR estimates for the remaining ones. We used funnel plots and Egger’s test to examine potential publication bias. We defined statistical significance as a *p*-value of < 0.05, except for the determination of publication bias, for which we used a *p* value of < 0.10.

## Results

### Literature search results

**Figure 1:**
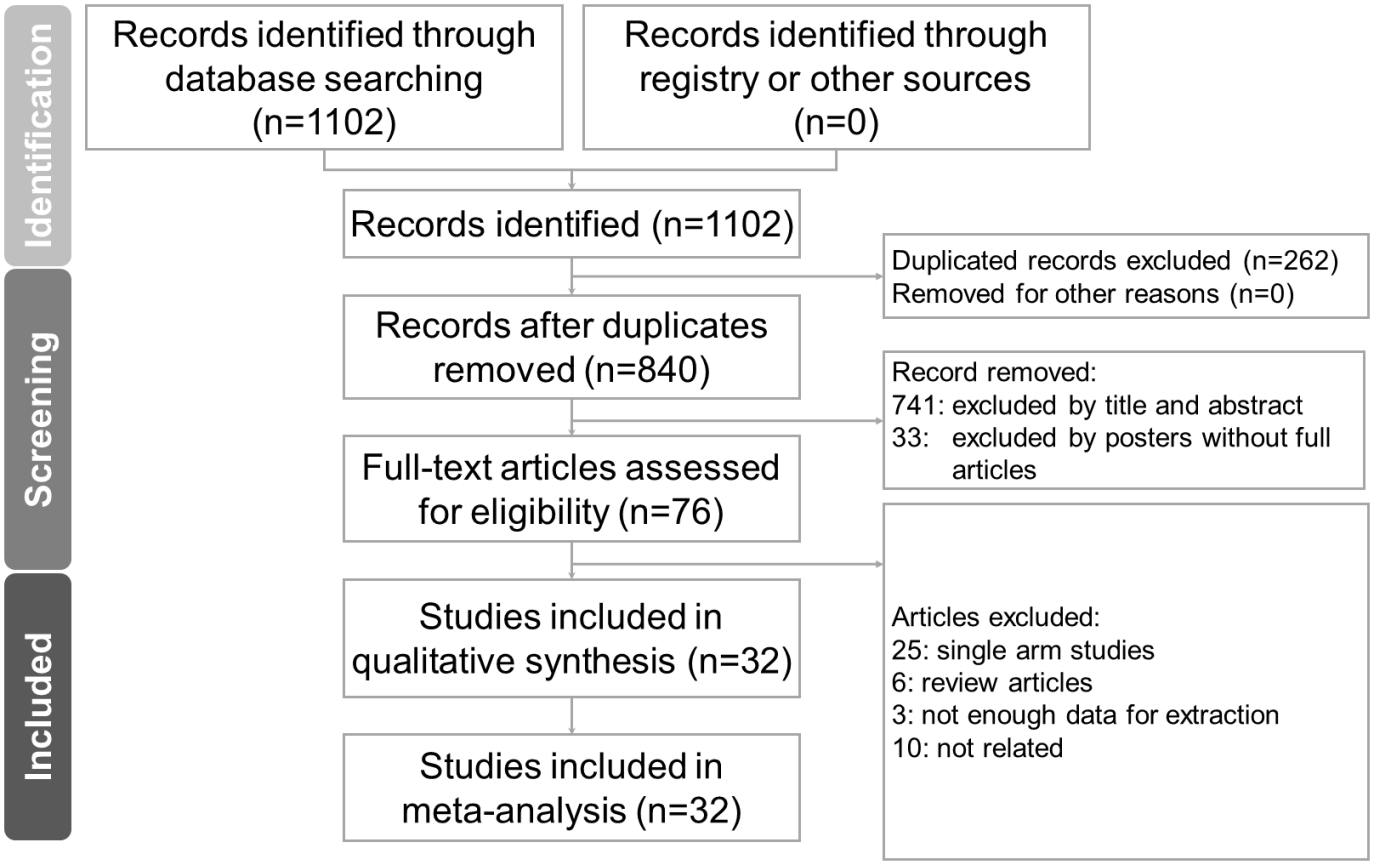
PRISMA (Preferred Reporting Items for Systematic Reviews and Meta-Analysis) flowchart of article selection process.

The literature search identified 840 non-duplicate references for a review of their titles and abstracts. After removing references violating the inclusion criteria, we included 76 studies for meticulous evaluation (**Figure 1**). We excluded 25 of those studies due to their single arm design, 6 review articles, 3 studies which had insufficient data for extraction, and 10 studies which did not meet our outcome of interest. The final quantitative analysis included 32 studies.

### Characteristics of included studies

**Table 1** lists the main characteristics of the 32 studies. In total, the studies included 9,631 patients, of which 3,941 (40.9%) were in a TBP group and 5,690 (59.1%) were in a non-TBP group. Only 5 (15.6%) prospective randomized studies were identified, and all the others (84.4%) employed a retrospective observational methodology. Most studies enrolled patients within the past 20 years and all studies were published within the past 10 years. There were 13 studies which evaluated ppPFS (1,758 patients) (17–20, 23–27, 30, 31, 36, 37), 20 studies which evaluated ppOS (8,271 patients) (13, 15–17, 19, 21, 23, 24, 27, 28, 30, 32–37, 39, 41, 44), and 12 studies evaluated OS from initiation of drugs (1,579 patients) (14, 15, 18, 22, 25, 32, 34, 38, 40–43). The drugs provided to the TBP groups fell into four categories: 14 studies used EGFR TKIs (43.8%) (13, 14, 17–19, 22, 23, 25, 26, 29, 30, 32, 36, 44), 4 employed ALK TKIs (12.5%) (15, 16, 34, 35), 10 studies used immunotherapy (31.3%) (28, 31, 33, 37–43), and 4 articles used anti-angiogenesis (12.4%) (20, 21, 24, 27).

**Table 1:** Summaries of characteristics of included studies.

				Number of participants		TBP group			Non-TBP group	outcome		
First author, year	Study period	Region	Study design	TBP	Non-TBP	Intervention drugs of TBP	Classification of intervention drugs	Add-on therapy		ppPFS	ppOS	OS from initiation of drugs
Faehling et al., 2013 (13)	2004-2011	Europe	Retrospective, observational	25	16	Erlotinib	EGFR TKI	with	Other and None		v	
Nishino et al., 2013 (14)	2002-2010	Asia	Retrospective, observational	93	242	Iressa	EGFR TKI	with	Other and None			v
Ou et al., 2014 (15)	-2012	Worldwide	Retrospective, observational	120	74	crizotinib	ALK TKI	without	Other and None		v	v
Chiari et al., 2015 (16)	2010-2015	Europe	Retrospective, observational	7	22	crizotinib/2nd G TKI	ALK TKI	with	Other		v	
HALMOS et al., 2015 (17)	2008-2012	America	Prospective, randomized	22	24	tarceva/tarceva+chemo	EGFR TKI	with	Other	v	v	
Auliac et al., 2016 (18)	2010-2012	Europe	Retrospective, observational	50	73	Iressa or Tarceva	EGFR TKI	with	Other and None	v		v
Schuler et al., 2016 (19)	2010-2011	worldwide	Prospective, randomized	134	68	afatinib+paclitazol vs chemo	EGFR TKI	with	Not M	v	v	
Higashiguchi et al., 2016 (20)	2007-2014	Asia	Retrospective, observational	23	49	avastin+chemo vs chemo	anti-angiogenesis	with	Other	v		
Leon et al., 2016 (21)	2006-2009	America	Prospective, observational	351	1007	avastin+chemo vs chemo	anti-angiogenesis	with	Other and None		v	
Moiseyenko et al., 2016 (22)	2006-2009	Europe	Retrospective, observational	21	49	Iressa	EGFR TKI	with	Other and None			v
Song et al., 2016 (23)	2011-2013	Asia	Retrospective, observational	38	92	Iressa or Tarceva +chemo	EGFR TKI	with	Other	v	v	
Takeda et al., 2016 (24)	2011-2013	Asia	Prospective, randomized	50	50	avastin+taxotere vs taxotere	anti-angiogenesis	with	Other	v	v	
WANG et al., 2016 (25)	2009-2014	Asia	Retrospective, observational	33	11	tarceva/Iressa	EGFR TKI	without	Other and None	v		v
Ding et al., 2017 (26)	2009-2015	Asia	Retrospective, observational	79	91	Iressa+chemo vs chemo	EGFR TKI	with	Other	v		
Mok et al., 2017 (44)	2012-2015	Worldwide	Prospective, randomized	133	132	Iressa+chemo vs chemo	EGFR TKI	with	Other		v	
Gridelli et al., 2018 (27)	2011-2015	Worldwide	Prospective, randomized	245	240	avastin+ standard care vs standard	anti-angiogenesis	with	Other	v	v	
Gandara et al., 2018 (28)	2014-2016	Worldwide	Retrospective, observational	168	94	atezolizumab	immunotherapy	without	Other		v	
Le et al., 2018 (29)	2014-2017	America	Retrospective, observational	47	26	tagrisso	EGFR TKI	with	Other and None			
Mehlman et al., 2019 (30)	2015-2018	Europe	Retrospective, observational	48	62	tagrisso	EGFR TKI	with	Other	v	v	
Metro et al., 2019 (31)	2017-2019	Europe	Retrospective, observational	18	42	pembrolizumab vs chemo	immunotherapy	with	Other	v		
Mu et al., 2019 (32)	2017-2018	Asia	Retrospective, observational	39	26	tagrisso	EGFR TKI	with	Other and None		v	v
Ricciuti et al., 2019 (33)	2013-2017	Europe	Retrospective, observational	60	116	nivolumab	immunotherapy	Not M	Other and None		v	
Xing et al., 2019 (34)	2013-2017	Asia	Retrospective, observational	140	121	crizotinib	ALK TKI	with	Other and None		v	v
Zhao et al., 2019 (35)	2013-2016	Asia	Retrospective, observational	19	15	crizotinib vs second G TKI	ALK TKI	with	other		v	
Cortellini et al., 2020 (36)	2015-2019	Europe	Retrospective, observational	50	41	tagrisso	EGFR TKI	with	other	v	v	
Ge et al., 2020 (37)	2015-2019	Asia	Retrospective, observational	39	86	Immunotherapy (mono or combination)	immunotherapy	Not M	Other and None	v	v	
Liang et al., 2020 (38)	2018-2019	Asia	Retrospective, observational	10	20	immunotherapy	immunotherapy	Not M	Other and None			v
Stinchcombe et al., 2020 (39)	2018-2019	America	Retrospective, observational	1668	2555	immunotherapy (mono)	immunotherapy	Not M	Other and None		v	
Won et al., 2020 (40)	2016-2018	Asia	Retrospective, observational	67	67	immunotherapy	immunotherapy	Not M	Other and None			v
Enomoto et al., 2021 (41)	2015-2018	Asia	Retrospective, observational	28	46	nivolumab	immunotherapy	without	Other		v	v
Heo et al., 2021 (42)	2011-2018	Asia	Retrospective, observational	16	25	Immunotherapy (80% mono)	immunotherapy	without	Other			v
Xu et al., 2021 (43)	2016-2020	Asia	Retrospective, observational	100	108	Immunotherapy (mono or combination)	immunotherapy	with	Other and None			v

TBP, treatment beyond progression; ppPFS, post progression progression-free survival; ppOS, post progression overall survival; OS from initiation of drugs, overall survival from initiation of drugs; EGFR, epidermal Growth Factor Receptor; ALK, anaplastic lymphoma kinase; TKI, tyrosine kinase inhibitors; G, generation; M, mention.

### Risk of bias

The risk of bias assessment for the 27 non-randomized studies used RoBANs (**Supplemental Table 1**). Performance bias and reporting bias were low in all studies. Only 10 studies had a low risk of detection bias and the remaining 17 studies were judged as unclear or at high risk of inadequate blinding of outcome assessments. Selection and attrition bias were low in most studies. Bias due to confounding variables were high in 10 studies, unclear in 1 study, and low in the remaining 16 studies. **Supplemental Table 2** shows risk of bias assessment using RoB 2 for five randomized studies. Two studies were rated as having “high risk of bias,” two as “some concerns,” and one as “low risk of bias.”

### Primary analysis

**Figure 2:**
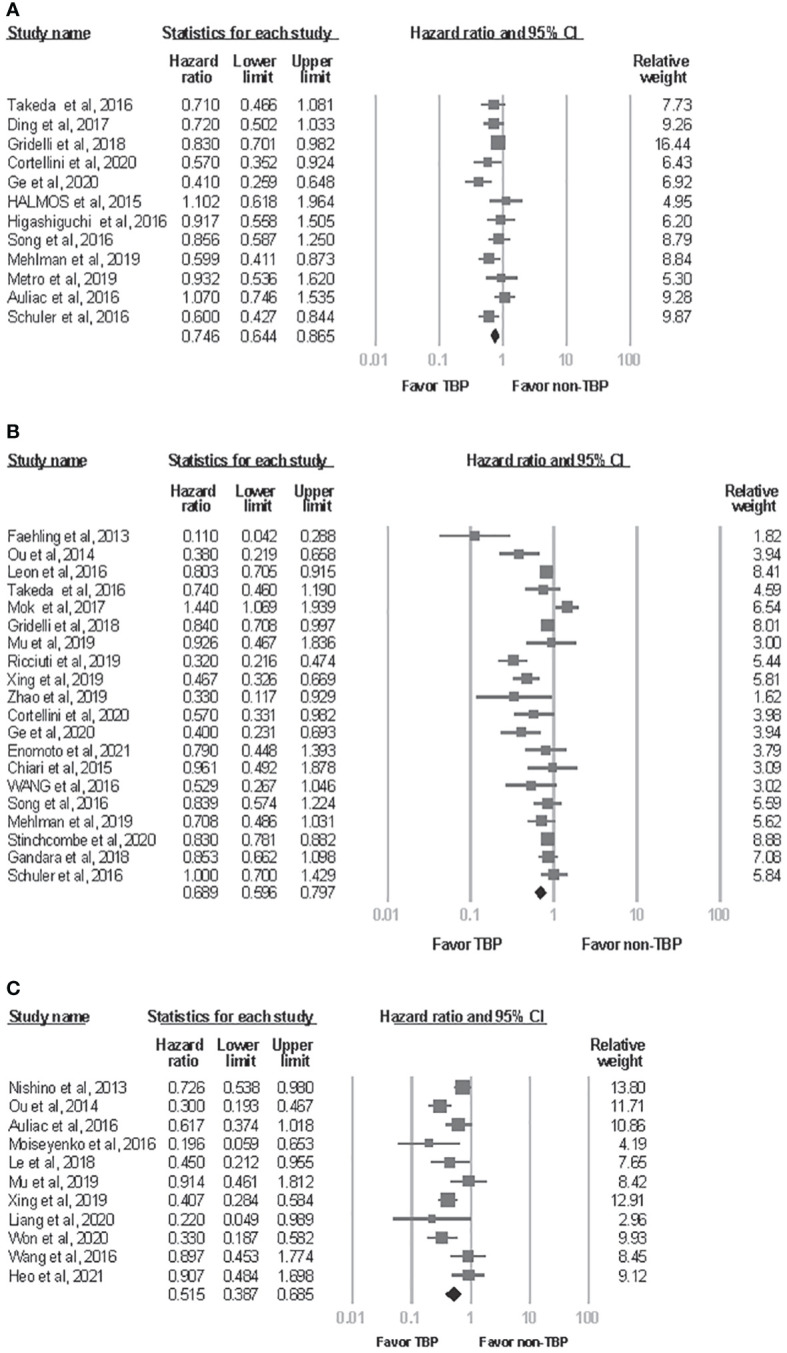
Forest plot of meta-analysis for effects of treatment beyond progression on survival outcome of NSCLC patients. **(A)** post progression progression-free survival. **(B)** post progression overall survival. **(C)** overall survival from initiation of drugs. CI, confidence interval; TBP, treatment beyond progression.

**Figure 3:**
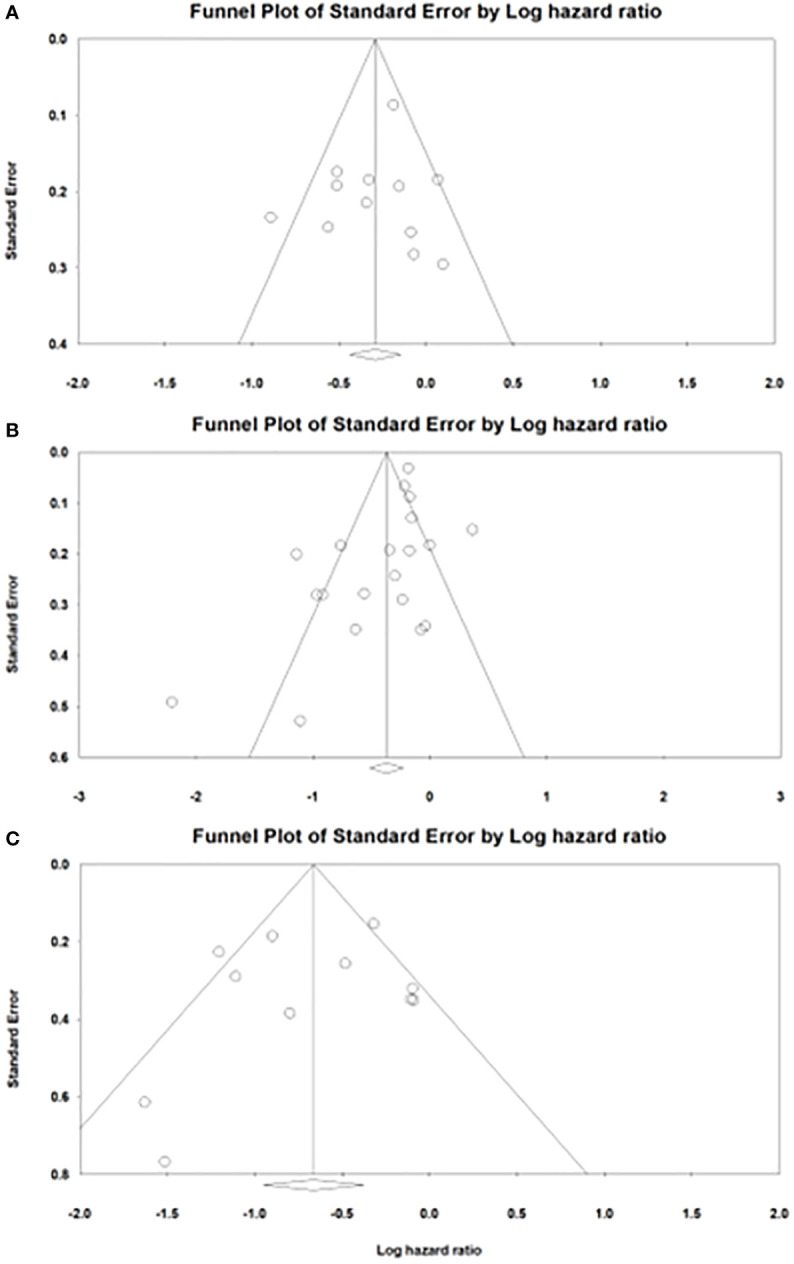
Funnel plots of publication bias in analysis of **(A)** post progression progression-free survival. **(B)** post progression overall survival. **(C)** overall survival from initiation of drugs.

Meta-analysis of the available literature revealed a statistically significant advantage for patients who received TBP compared with those who did not in ppPFS, ppOS, and OS from initiation of drugs (ppPFS: HR, 0.746; 95% CI, 0.644-0.865; *P*<0.001; ppOS: HR, 0.689; 95% CI, 0.596-0.797; *P*<0.001; OS from initiation of drugs: HR, 0.515; 95% CI, 0.387-0.685; P<0.001)(**Figure 2**). Statistically significant between-study heterogeneity was noted among results of ppOS (*I* ^2 =^ 77.5%, P<0.001) and OS starting from initiation of drugs (*I* ^2 =^ 63.436, *P*=0.002), but not in ppPFS (*I* ^2 =^ 43.4%, *P*=0.053). In sensitivity analysis, exclusion of any single study did not essentially vary the overall results of the primary analysis. Significant publication bias was detected in analysis of ppOS (Egger’s test, ppOS: *P*=0.041), but not in ppPFS and OS from initiation of drugs (Egger’s test, ppPFS: *P*=0.560; OS from initiation of drugs: *P*=0.550) (**Figure 3**).

### Subgroup analysis

Subgroup analysis according to classification of the TBP drugs, treatment of the non-TBP group, whether add-on therapy was allowed in the TBP group, and region are shown in **Table 2** and **Supplemental Figure 1A**-**3C**, respectively. Subgroup analysis of the classification of TBP drugs revealed that EGFR TKIs resulted in significantly improved ppPFS (HR, 0.751; 95% CI, 0.617-0.914; I^2^, 41.2%) and OS from initiation of drugs (HR, 0.660; 95% CI, 0.498-0.875; I^2^, 28.0%), but not ppOS (HR, 0.713; 95% CI, 0.493-1.031; I^2^, 79.9%). ALK TKIs showed significantly improved ppOS (HR, 0.496; 95% CI, 0.335-0.735; I^2^, 43.8%) and OS from initiation of drugs (HR, 0.359; 95% CI, 0.268-0.482; I^2^, 8.8%). Immunotherapy produced significantly improved ppOS (HR, 0.612; 95% CI, 0.432-0.867; I^2^, 86.1%), but not ppPFS (HR, 0.609; 95% CI, 0.403-0.815; I^2^, 80.1%) or OS from initiation of drugs (HR, 0.455; 95% CI, 0.198-1.048; I^2^, 70.0%). Anti-angiogenesis agents resulted in significantly improved ppPFS (HR, 0.821; 95% CI, 0.708-0.953; I^2^, 0%) and ppOS (HR, 0.813; 95% CI, 0.734-0.899; I^2^, 0%).

**Table 2:** Differences of survival outcomes by subgroups.

	No. of reports	HR	95% CI	*P*	I^2^(%)	*P* Value forheterogeneity
ppPFS						
Classification of TBP intervention drugs						
EGFR TKI	7	0.751	0.617-0.914	0.004	41.220	0.116
Anti-angiogenesis	3	0.821	0.708-0.953	0.010	0.000	0.717
Immunotherapy	2	0.609	0.403-0.815	0.227	80.125	0.025
Treatment of non-TBP treatment						
Other	10	0.767	0.688-0.854	<0.001	0.000	0.437
Other and None	2	0.670	0.262-1.715	0.403	90.390	0.001
Region						
America	1	1.102	0.618-1.964	0.742	0.000	1.000
Asia	5	0.701	0.543-0.906	0.007	46.554	0.112
Europe	4	0.766	0.552-1.063	0.111	56.237	0.077
Worldwide	2	0.731	0.536-0.997	0.048	64.302	0.094
ppOS						
Classification of TBP intervention drugs						
EGFR TKI	8	0.713	0.493-1.031	0.072	79.945	<0.001
ALK TKI	4	0.496	0.335-0.735	<0.001	43.782	0.149
Anti-angiogenesis	3	0.813	0.734-0.899	<0.001	0.000	0.850
Immunotherapy	5	0.612	0.432-0.867	0.006	86.056	<0.001
Non-TBP treatment						
Other	12	0.808	0.677-0.964	0.005	59.000	0.006
Other and None	8	0.531	0.407-0.694	<0.001	87.438	<0.001
Add-on therapy in the TBP group						
With add-on	12	0.744	0.602-0.919	0.006	76.523	<0.001
Without add-on	4	0.636	0.429-0.943	0.024	61.592	0.050
Region						
America	2	0.825	0.781-0.872	<0.001	0.000	0.649
Asia	8	0.616	0.483-0.786	<0.001	38.976	0.119
Europe	5	0.456	0.262-0.794	0.005	81.501	0.000
Worldwide	5	0.874	0.652-1.172	0.369	80.180	0.000
OS from initiation of drugs						
Classification of TBP intervention drugs						
EGFR TKI	6	0.660	0.498-0.875	0.004	28.003	0.225
ALK TKI	2	0.359	0.268-0.482	<0.001	8.790	0.295
Immunotherapy	3	0.455	0.198-1.048	0.064	69.962	0.036
Add-on therapy in the TBP group						
With add-on	6	0.553	0.400-0.764	<0.001	55.196	0.048
Without add-on	3	0.606	0.273-1.345	0.218	82.450	0.003
Region						
America	1	0.450	0.212-0.955	0.037	0.000	1.000
Asia	7	0.595	0.421-0.841	0.003	63.437	0.012
Europe	2	0.398	0.134-1.187	0.098	66.407	0.084
Worldwide	1	0.300	0.193-0.467	0.000	0.000	1.000

HR, hazard ratio; CI, confidence interval; TBP, treatment beyond progression; ppPFS, post progression progression-free survival; ppOS, post progression overall survival; OS from initiation of drugs, overall survival from initiation of drugs; EGFR, epidermal Growth Factor Receptor; ALK, anaplastic lymphoma kinase; TKI, tyrosine kinase inhibitors.

Subgroup analysis of the non-TBP treatment group showed significantly improved ppPFS in the Other treatment group (HR, 0.767; 95% CI, 0.688-0.854; I^2^, 0%), but not in the Other and None treatment group(HR, 0.670; 95% CI, 0.262-1.715; I^2^, 90.4%). Improved ppOS was also evident (Other treatment group: HR, 0.808; 95% CI, 0.677-0.964; I^2^, 59.0%; Other and None treatment group: HR, 0.531; 95% CI, 0.407-0.694; I^2^, 87.4%). Subgroup analysis to assess results of add-on therapy with TBP showed that the With add-on group had significantly improved ppOS (HR, 0.744; 95% CI, 0.602-0.919; I^2^, 76.5%) and OS from initiation of drugs (HR, 0.553; 95% CI, 0.400-0.764; I^2^, 55.2%). The Without add-on group likewise demonstrated significantly improved ppOS (HR, 0.636; 95% CI, 0.429-0.943; I^2^, 61.6%), but showed no benefit for OS from initiation of drugs (HR, 0.606; 95% CI, 0.273-1.345; I^2^, 82.5%). Subgroup analysis of region demonstrated significantly improved ppPFS, ppOS and OS from initiation of drugs in the Asia group but less consistent results of other subgroups, which might be due to limited number of studies or high heterogeneity. When analyzing ppOS and OS from initiation of drugs, subgroup analysis according to classes of drugs decreased heterogeneity between studies.

## Discussion

To the best of our knowledge, this is the first systematic review and meta-analysis to focus on whether or not the TBP treatment strategy provided survival benefit for NSCLC patients. Our findings suggest that TBP may improve ppPFS, ppOS and OS from initiation of drugs.

In recent years, immunotherapy, mainly consisting of checkpoint inhibitors including anti-programmed death 1 and anti–programmed death-ligand 1, has dramatically changed cancer treatment paradigms. Immune checkpoint inhibitors stimulate the immune system to attack tumors instead of targeting tumor cells directly, exhibiting different patterns of response to immunotherapy (45). These include alterations in tumor biology reflecting anticancer efficacy following initial radiographic PD (46). Because uncertainty about whether immunotherapy was discontinued and late benefit from treatment continuation among some patients, most clinical trials of immunotherapies permit treatment beyond RECIST-defined PD as long as performance status remains acceptable, the patient provides consent, no serious toxic effects, and no impending end organ damage is observed (47, 48).

Immunotherapy TBP may be a rational treatment choice for the following reasons. First, about 0.6 to 5.8% patients with NSCLC may initially experience increased size of tumor lesions during immunotherapy treatment, followed by a delayed partial response (49). This phenomenon is called “pseudo-progression,” and possibly results from infiltration and recruitment of lymphocytes in the tumor (50). Second, the interaction between the tumor and the immune system may be a long term process which could possibly result in undulant clinical effects, such as undulating tumor growth and shrinkage (48). Third, radiotherapy and chemotherapy may have synergistic effects when combined with immunotherapy *via* the release of tumor antigen, causing a proinflammatory environment and resulting in activation and clonal expansion of T cells (51, 52). Forth, lesion-level heterogeneity at the time of RECIST-defined PD was common in immunotherapy-treated patients and they these patients may demonstrate ongoing disease control in a subset of tumor sites (53).

In our meta-analysis, TBP significantly prolonged ppOS without statistically significant benefit for ppPFS and OS from initiation of drugs. Enomoto et al. demonstrated no significant difference in ppOS between TBP and the other treatment group. The definition of TBP (nivolumab ≥ 2 weeks after first PD using RECIST v1.1) may explain the less favorable results obtained with nivolumab beyond progression in this study compared with other studies, such as Ricciuti et al. (nivolumab ≥ 6 weeks after first PD using RECIST v1.1) (33, 41). Metro et al. showed no significant difference in ppPFS after comparing pembrolizumab beyond progression and salvage chemotherapy (31). Despite the study’s small sample size, pembrolizumab TBP could be beneficial in select patients. Among the nine patients in the TBP group with the addition of local ablative radiotherapy and PD in no more than two organ sites, the ppPFS rates at 6 and 12 months were high at 88.9% and 71.1%, respectively. Based on previous studies, TBP with immunotherapy may be beneficial in specific circumstances, such as oligo-progression, PD without new lesion, in patients with good performance status, and with add on treatment (41, 43, 53).

For many years, first and second generation EGFR TKIs represented milestones of first line treatment in NSCLC patients with EGFR mutations (54). Osimertinib, a third generation TKI, could overcome treatment resistance acquired after use of first line TKIs, such as T790M. In first line settings, T790M showed better efficacy compared to first and second generation TKIs (55, 56). However, most patients receiving EGFR TKIs eventually developed TKIs resistant progressions. Moreover, EGFR-mutant lung cancer patients showed poor responses to immunotherapy treatment (57). To achieve better survival among EGFR mutant lung cancer patients, we need to prolong treatment duration with EGFR TKIs. In clinical practice, continuing EGFR TKIs still benefited some patients with EGFR mutation who developing acquired resistance to Erlotinib or gefitinib, suggesting that part of tumor cells remained sensitive to EGFR-TKIs (58).

Oxnard et al. explained this phenomenon *in vitro* using EGFR*-*mutant NSCLC cell lines; they pointed out that resistant tumors are likely a mixed components of EGFR-TKIs-sensitive and resistant cells (59). In our subgroup analysis, TBP with EGFR TKIs significantly prolonged ppPFS and OS from initiation of drugs but had no significant benefit in ppOS. Mok et al. and Ding et al. showed that gefitinib plus chemotherapy was not beneficial for patients with acquired resistance to first line gefitinib, however, patients with T790M negative tumors may be the select patients who can benefit from continuation of gefitinib beyond progression (26, 44). In previous studies, patients with gradual progression rather than dramatic progression, oligo-progressive disease, and added on therapy using local ablative treatments may also be among the select patients who benefit from TBP (32, 36, 60, 61).

Since the 2007 discovery of ALK rearrangement in NSCLC, tremendous strides have been made in the treatment of ALK positive NSCLC, best exemplified by the approval of six ALK TKIs (62). Our data show that TBP with ALK TKIs may further prolong ppOS and OS from initiation of drugs. Results from Chiari et al. revealed negative results of TBP; unsurprisingly, shifting to second generation ALK TKIs produced better ppPFS than TBP with first generation TKI, because second generation ALK TKIs may overcome some mechanisms of resistance to first generation ALK TKIs (16, 63). Therefore, a reasonable treatment strategy could be to maximize treatment duration of TBP with each line of ALK TKIs, then shifting to the next line ALK TKIs which impact resistance pathways produced by the previous line ALK TKIs.

Anti-angiogenesis agents, such as bevacizumab, which is a recombinant, humanized monoclonal antibody that targets vascular endothelial growth factor (VEGF), have been approved for treatment of non-squamous NSCLC in combination with chemotherapy, target therapies, and immunotherapy (64). Targeted action against angiogenesis can cause normalization or regression of existing tumor vasculature and the inhibition of new and recurrent tumor vessel growth (65). Furthermore, due to the multiple effects of VEGF on the tumor immune microenvironment, targeting VEGF with anti-angiogenesis agents enhances the anti-cancer immune response (66). Given the mechanism of action of anti-angiogenesis agents, there is a rationale for TBP with added-on TKIs or chemotherapy, on purpose to maintain an angiogenesis blockade (67). Our data supports that TBP with anti-angiogenesis agents prolongs ppPFS and ppOS. However, Takeda et al. reported no significant survival benefit of TBP with bevacizumab; subgroup analysis of their data revealed that patients whose disease progressed starting at least six months after the initiation of first-line chemotherapy and those who achieved a complete or partial response to first-line treatment gained more advantage from bevacizumab continuation (24). In another study that observed negative results of TBP, Higashiguchi et al. nonetheless claimed that they could not deny the possibility of the benefits of TBP with bevacizumab, because it was associated with a better response rate and the OS of the TBP group with bevacizumab looked slightly better than that of the non-TBP group (20).

The results of this study have some limitations. First, as in any meta-analysis, analysis of results of the study limited to the data reported by the authors. Precisely, some authors do not present the HRs, and we could only calculate HRs and the associated statistics based on the information given in the study report. Second, most of the studies were observational, not randomized controlled trials. Observational studies are likely to have greater potential biases than randomized studies because randomized studies adjust known and unknown confounders to balance across different groups. Therefore, we should always interpret results cautiously when observational studies are included in reviews and meta-analyses. Third, this meta-analysis did not include data on individual patients. As a result, it was not possible to adjust patient variables such Eastern Cooperative Oncology Group performance status, age, and race. Fourth, the between-study heterogeneity became lower but was still high after subgroup analysis. Factors that could potentially explain the heterogeneity may include the definition of TBP, which differed in each study; moreover, more than half of the studies did not provide one. Fifth, potential bias of recruitment may contribute to meaningful OS differences. Patients who were selected to TBP groups may have, to a varying degree, better condition such as performance status than those who were not.

## Conclusions

This study provides further evidence in support of TBP for NSCLC, however, these results require cautious interpretation. Currently, clinicians and patients are left with uncertainty about how best to deal with disease progression. Treatment decisions will continue to depend on many points, including the availability of other therapeutic agents, clinician’s instincts, and the patient’s evaluation of benefits and risks. Large, randomized, prospective controlled trials to investigate the efficacy of TBP in lung cancer treatment and the biomarkers to predict the populations who may benefit from TBP are warranted.

## Data availability statement

The raw data supporting the conclusions of this article will be made available by the authors, without undue reservation.

## Author contributions

W-KK and Y-JL designed the study. W-KK and Y-JL designed the statistical plan. W-KK and Y-JL performed the key analyses. W-KK and Y-JL generated and collected the data. C-FW assisted in data interpretation. W-KK wrote the manuscript. C-FW and Y-JL revised the manuscript. All authors contributed to the article and approved the submitted version.

## Conflict of interest

The authors declare that the research was conducted in the absence of any commercial or financial relationships that could be construed as a potential conflict of interest.

## Publisher’s note

All claims expressed in this article are solely those of the authors and do not necessarily represent those of their affiliated organizations, or those of the publisher, the editors and the reviewers. Any product that may be evaluated in this article, or claim that may be made by its manufacturer, is not guaranteed or endorsed by the publisher.

## Supplementary material

Supplementary Figure 1 Forest plot of subgroup analysis of association between post progression progression-free survival and **(A)** classification of treatment beyond progression (TBP) drugs. **(B)** treatment of the non-TBP group. **(C)** region.

Supplementary Figure 2 Forest plot of subgroup analysis of association between post progression overall survival and **(A)** classification of treatment beyond progression (TBP) drugs. **(B)** whether add-on therapy was allowed in the TBP group. **(C)** treatment of the non-TBP group. **(D)** region.

Supplementary Figure 3 Forest plot of subgroup analysis of association between overall survival from initiation of drugs and **(A)** classification of treatment beyond progression (TBP) drugs. **(B)** whether add-on therapy was allowed in the TBP group. **(C)** region.
