# Supplementary figures and images for: Treatment beyond progression in non-small cell lung cancer: A systematic review and meta-analysis
Source: Front Oncol. 2022 Nov 17;12:1023894. doi: 10.3389/fonc.2022.1023894 (PMC9713814; doi:10.3389/fonc.2022.1023894)

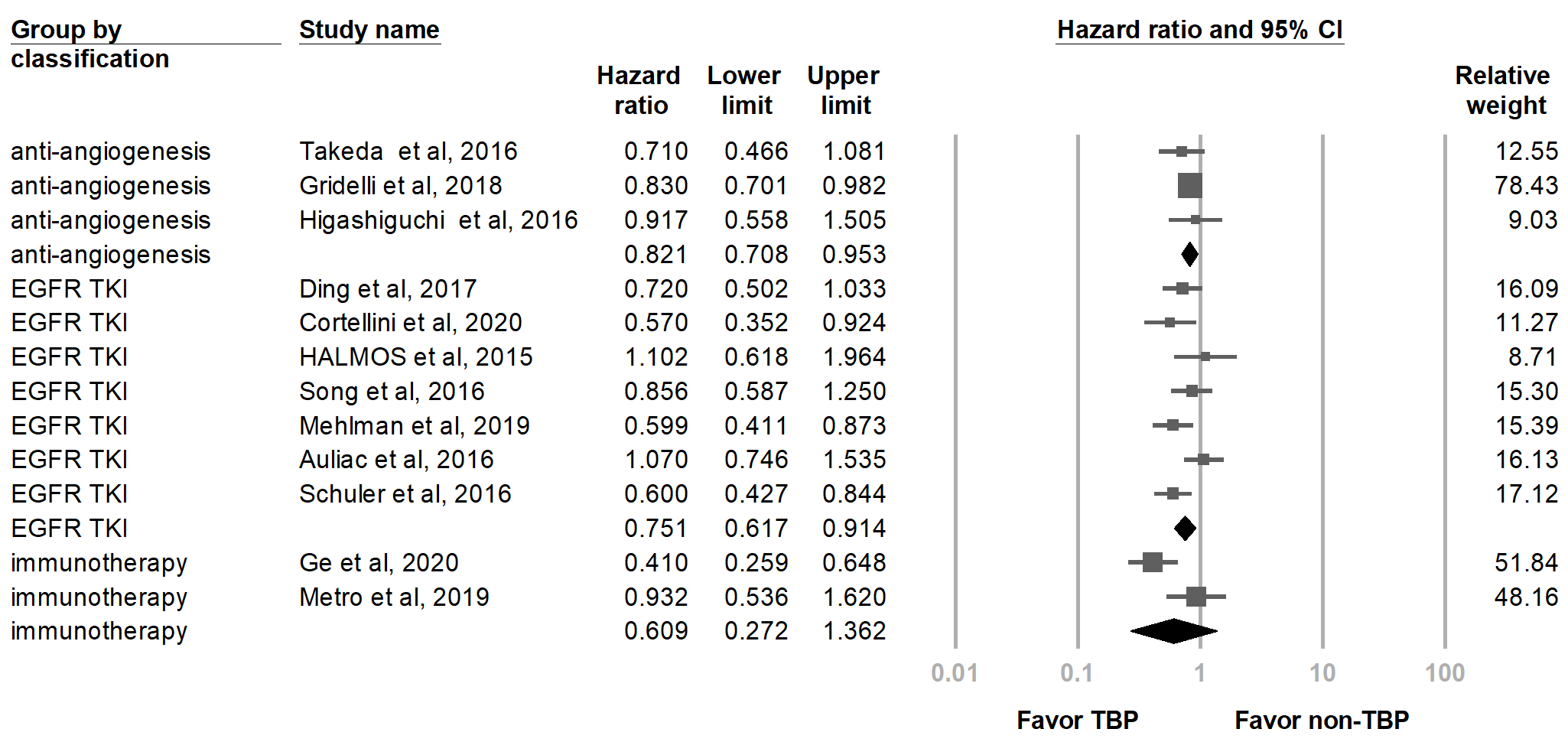

Supplement: Supplementary Figure 1 — Forest plot of subgroup analysis of association between post progression progression-free survival and (A) classification of treatment beyond progression (TBP) drugs. (B) treatment of the non-TBP group. (C) region. [file DataSheet_1.zip › Supplemental Figure 1A.TIF]

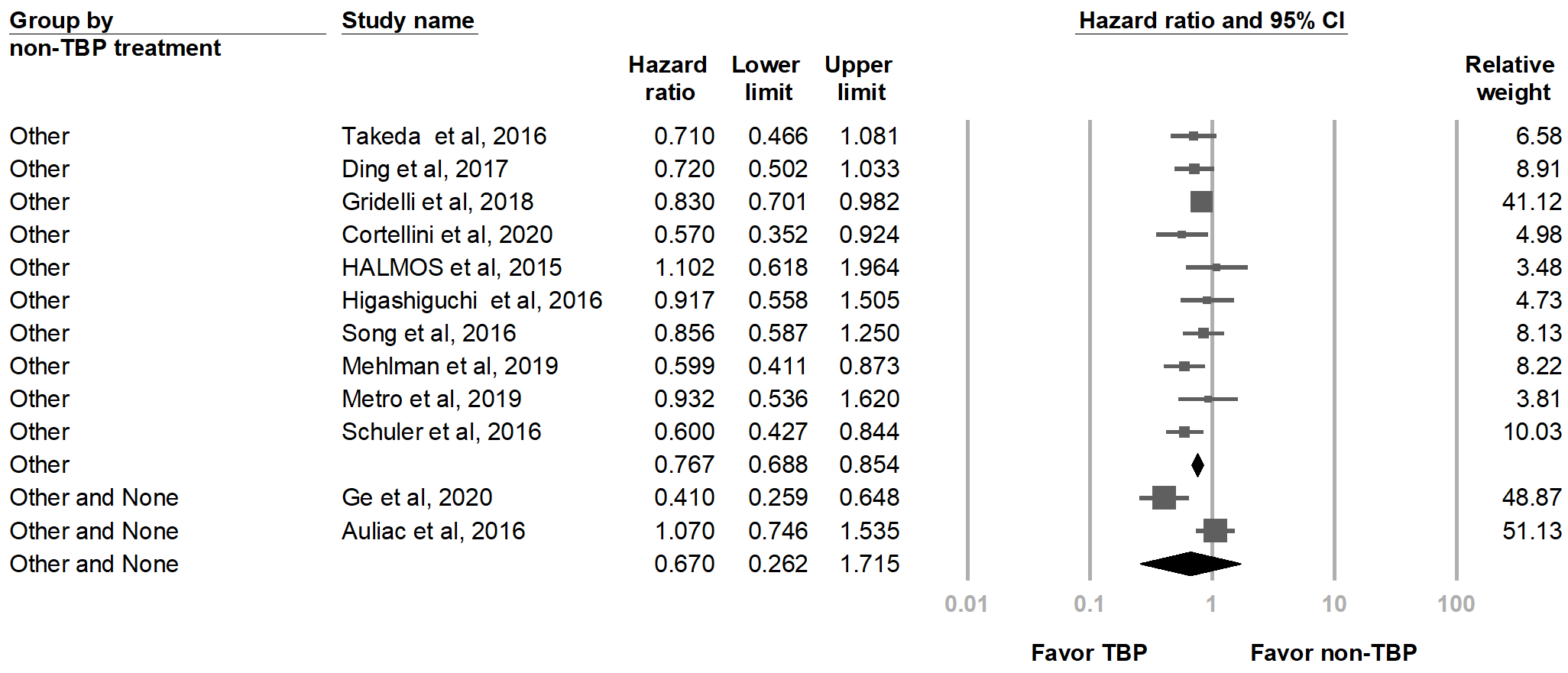

Supplement: Supplementary Figure 1 — Forest plot of subgroup analysis of association between post progression progression-free survival and (A) classification of treatment beyond progression (TBP) drugs. (B) treatment of the non-TBP group. (C) region. [file DataSheet_1.zip › Supplemental Figure 1B.TIF]

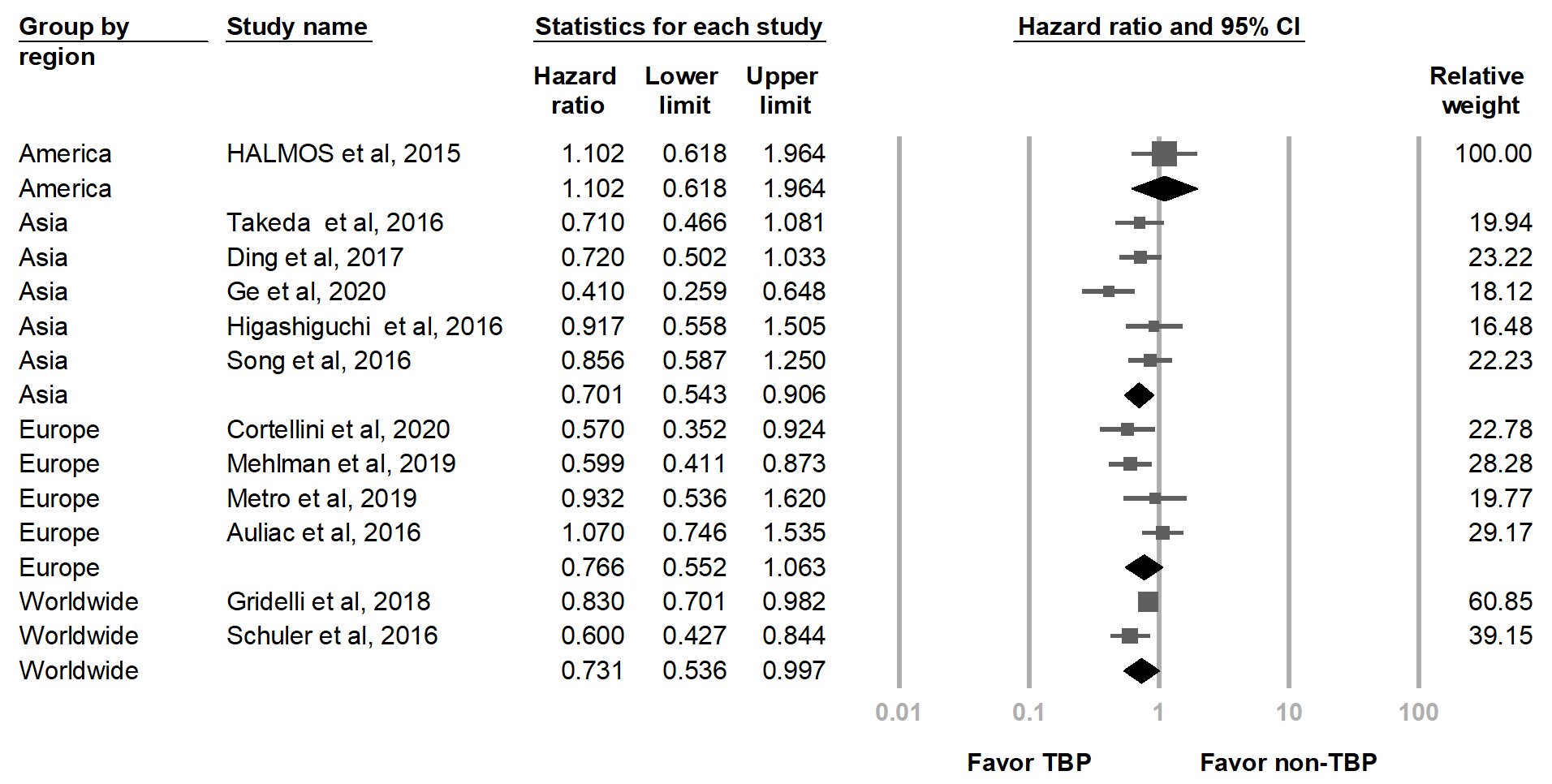

Supplement: Supplementary Figure 1 — Forest plot of subgroup analysis of association between post progression progression-free survival and (A) classification of treatment beyond progression (TBP) drugs. (B) treatment of the non-TBP group. (C) region. [file DataSheet_1.zip › Supplemental Figure 1C.tif]

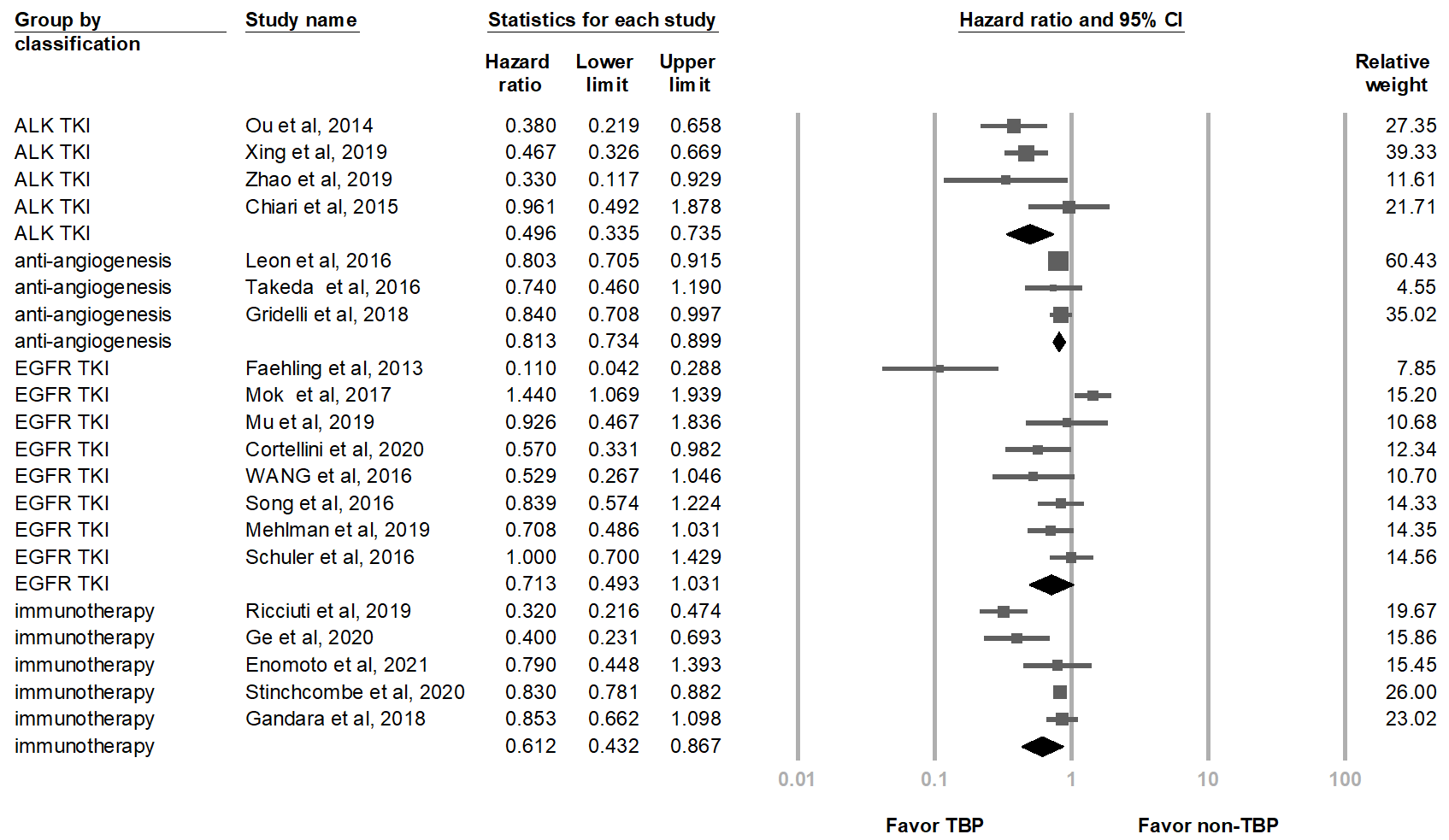

Supplement: Supplementary Figure 1 — Forest plot of subgroup analysis of association between post progression progression-free survival and (A) classification of treatment beyond progression (TBP) drugs. (B) treatment of the non-TBP group. (C) region. [file DataSheet_1.zip › Supplemental Figure 2A.tif]

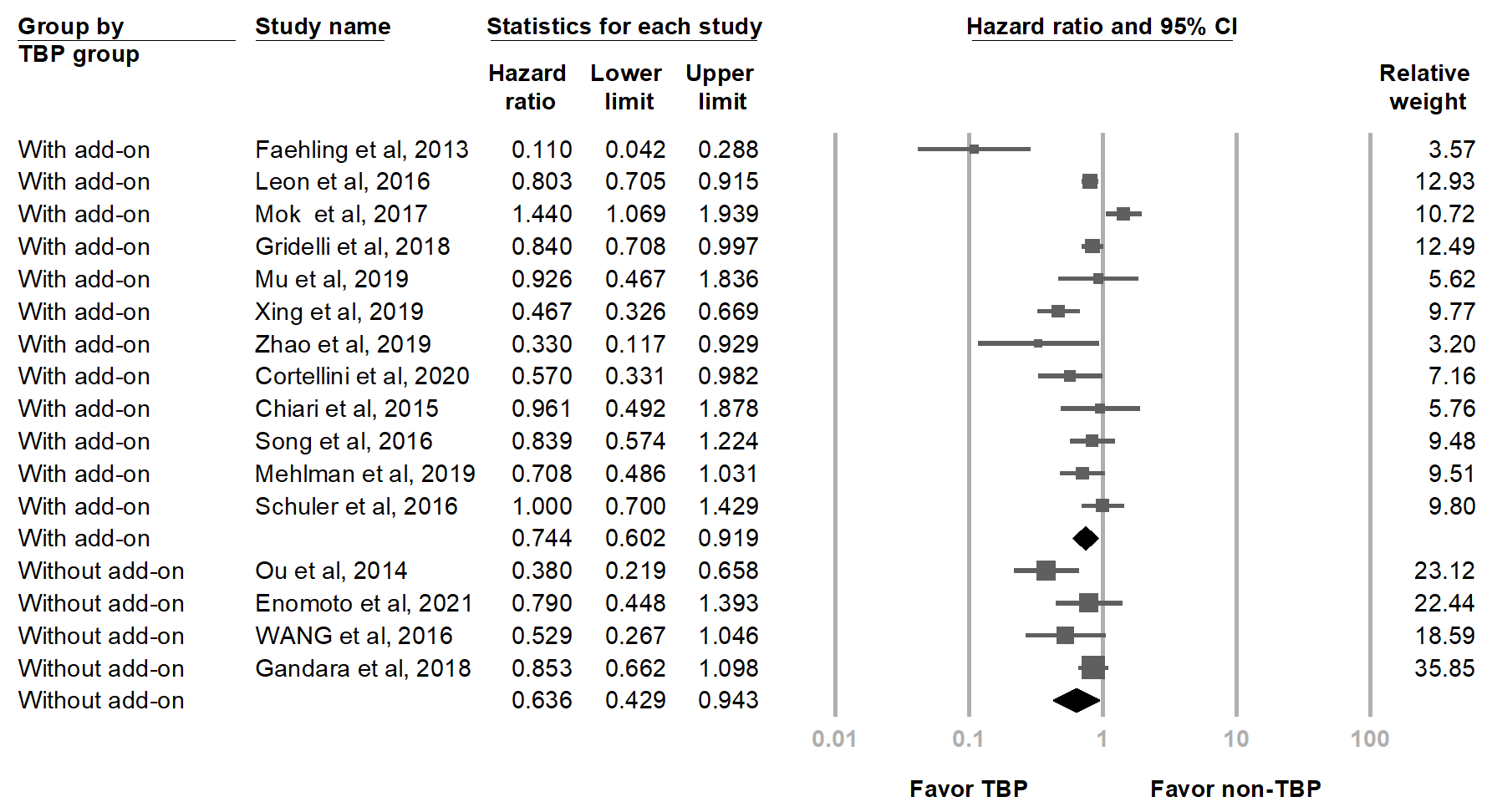

Supplement: Supplementary Figure 1 — Forest plot of subgroup analysis of association between post progression progression-free survival and (A) classification of treatment beyond progression (TBP) drugs. (B) treatment of the non-TBP group. (C) region. [file DataSheet_1.zip › Supplemental Figure 2B.tif]

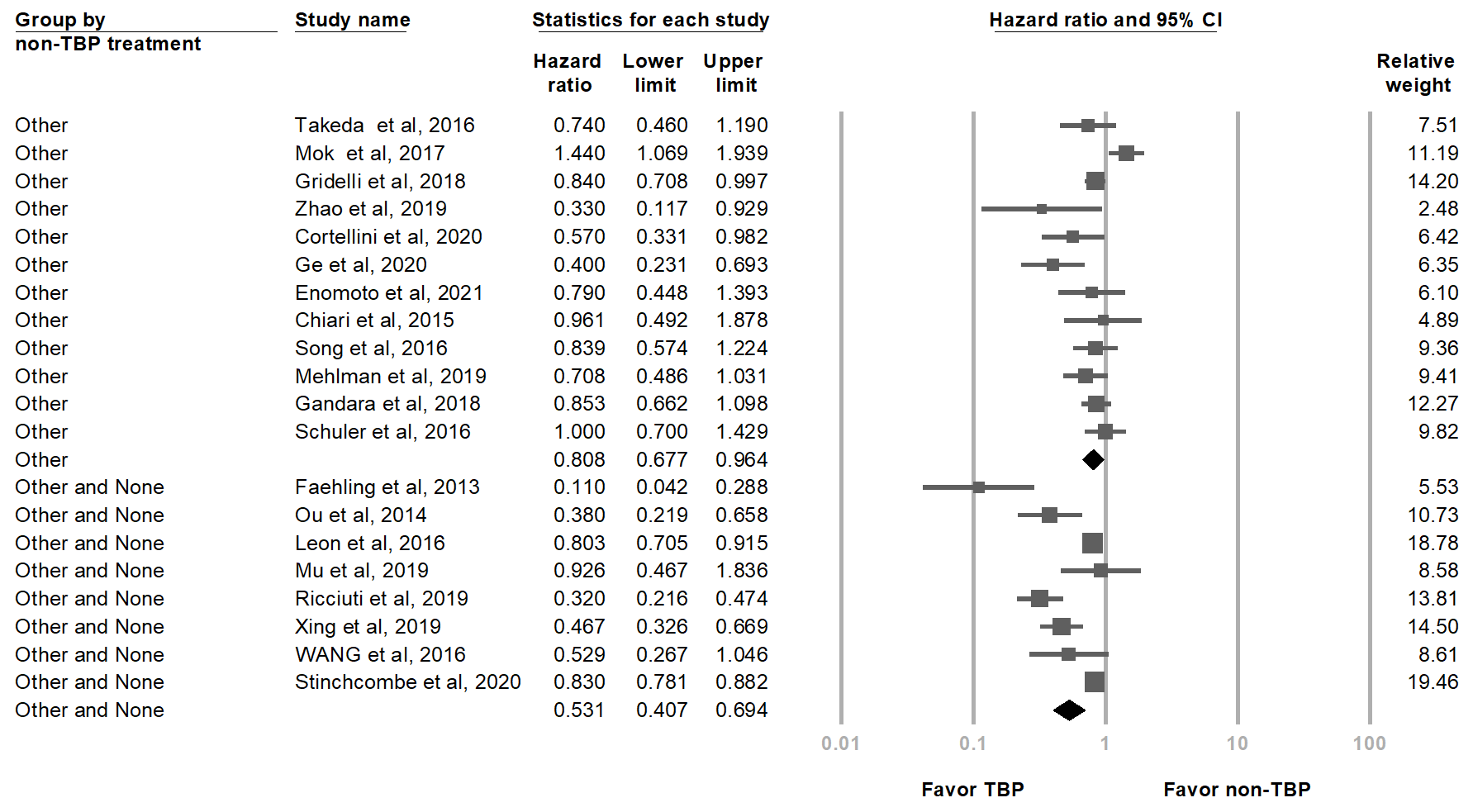

Supplement: Supplementary Figure 1 — Forest plot of subgroup analysis of association between post progression progression-free survival and (A) classification of treatment beyond progression (TBP) drugs. (B) treatment of the non-TBP group. (C) region. [file DataSheet_1.zip › Supplemental Figure 2C.tif]

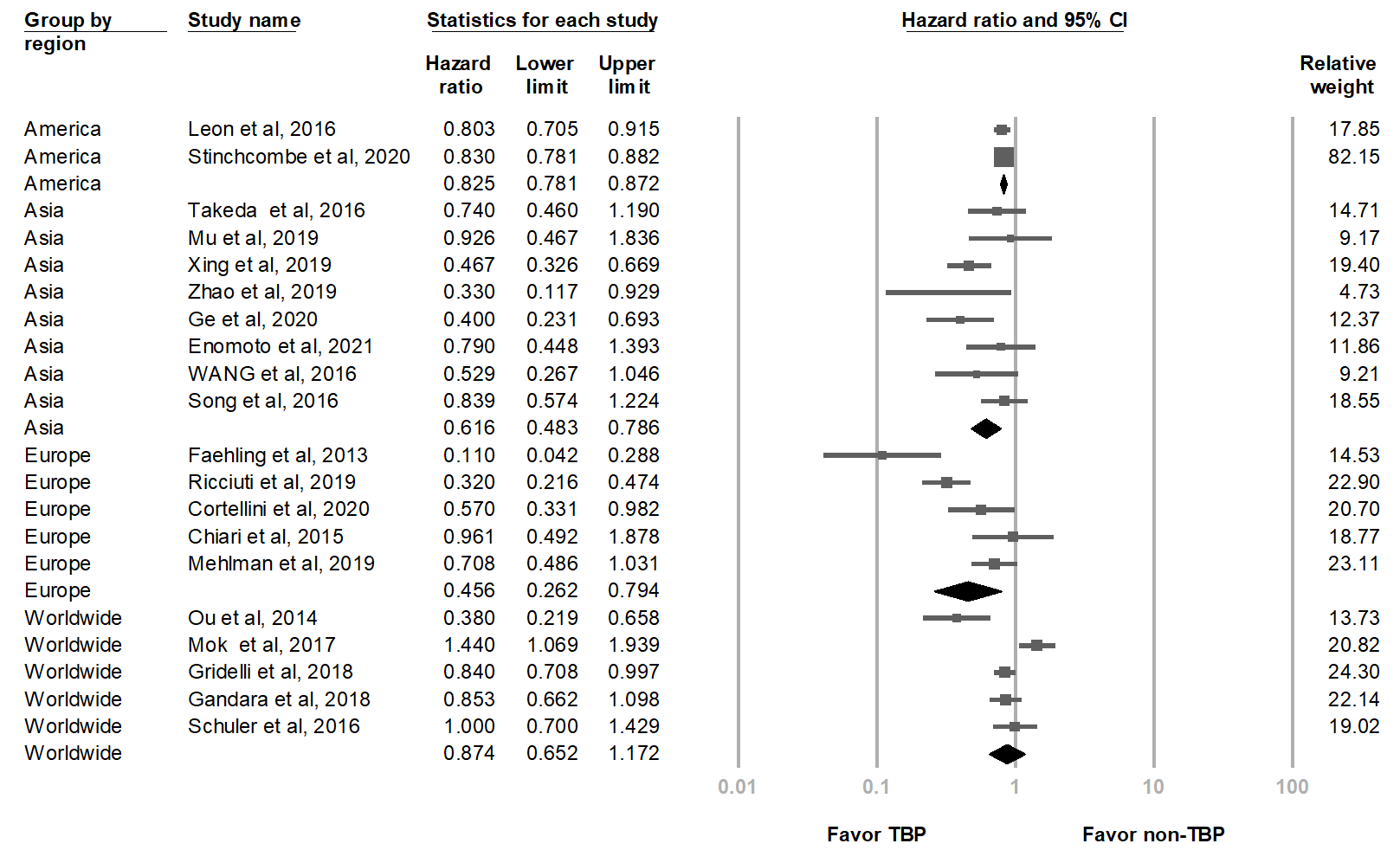

Supplement: Supplementary Figure 1 — Forest plot of subgroup analysis of association between post progression progression-free survival and (A) classification of treatment beyond progression (TBP) drugs. (B) treatment of the non-TBP group. (C) region. [file DataSheet_1.zip › Supplemental Figure 2D.tif]

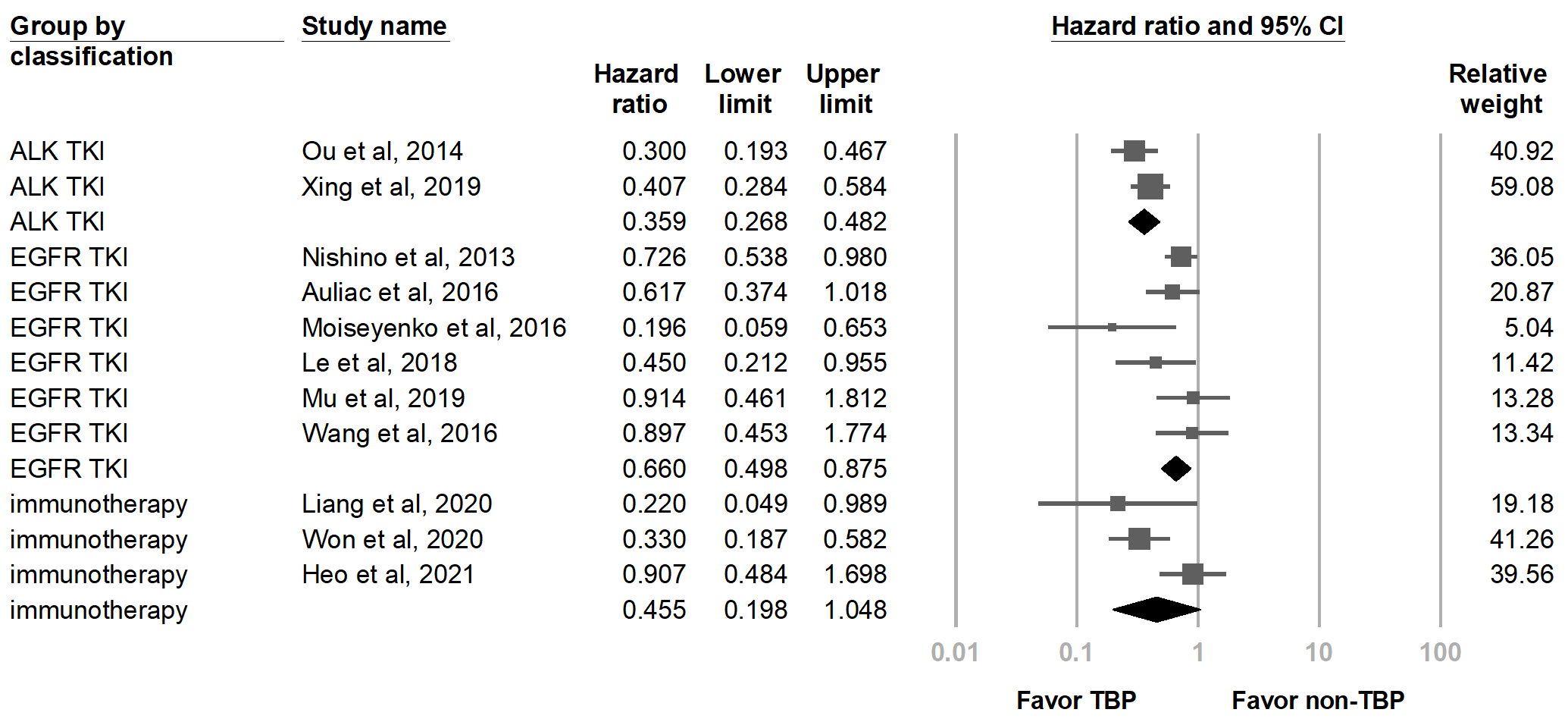

Supplement: Supplementary Figure 1 — Forest plot of subgroup analysis of association between post progression progression-free survival and (A) classification of treatment beyond progression (TBP) drugs. (B) treatment of the non-TBP group. (C) region. [file DataSheet_1.zip › Supplemental Figure 3A.tif]

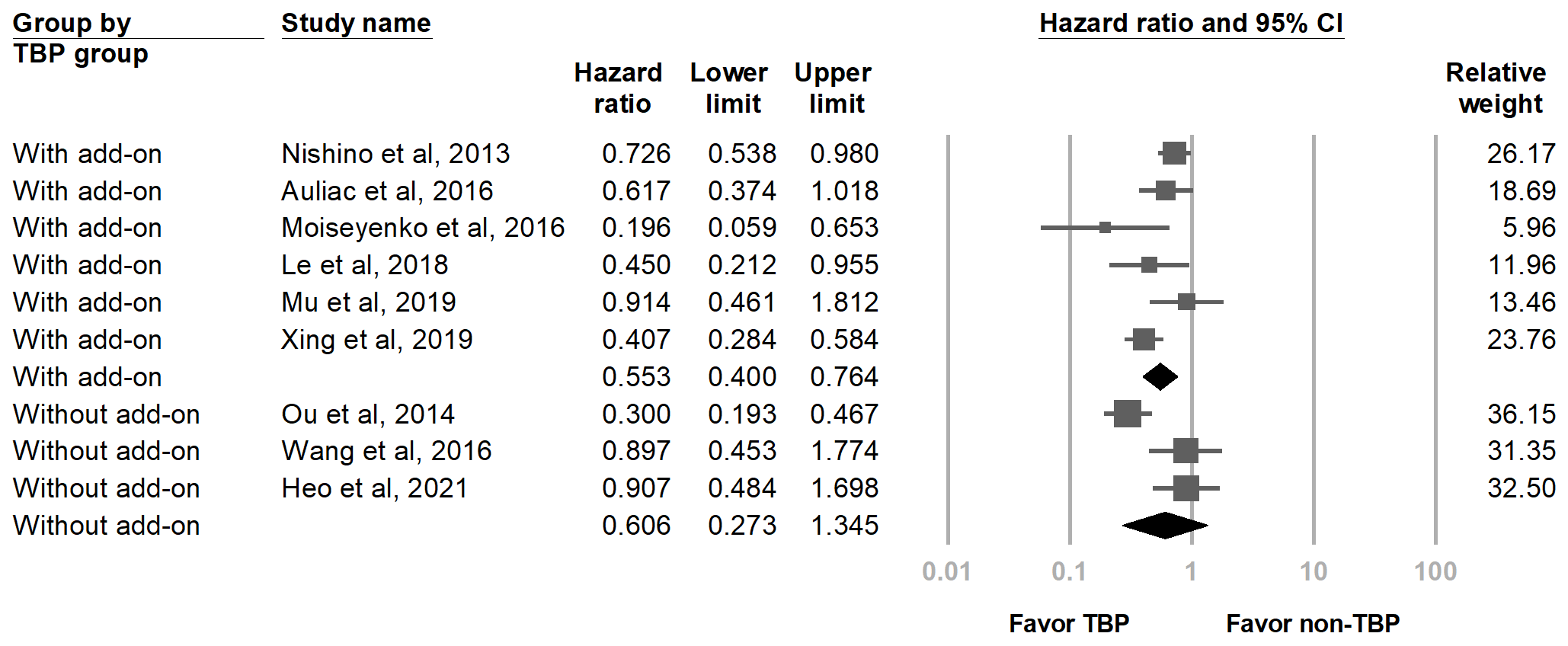

Supplement: Supplementary Figure 1 — Forest plot of subgroup analysis of association between post progression progression-free survival and (A) classification of treatment beyond progression (TBP) drugs. (B) treatment of the non-TBP group. (C) region. [file DataSheet_1.zip › Supplemental Figure 3B.tif]

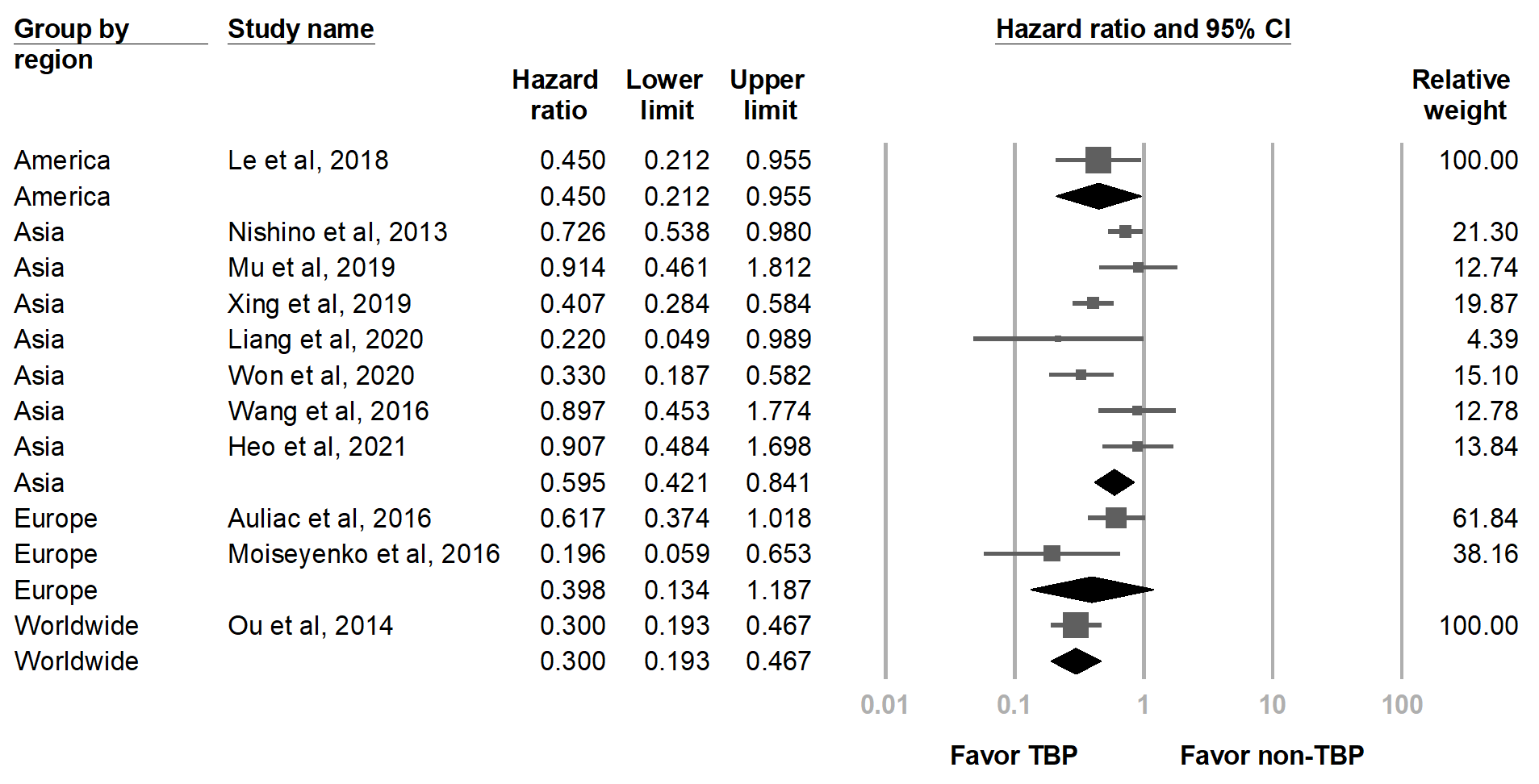

Supplement: Supplementary Figure 1 — Forest plot of subgroup analysis of association between post progression progression-free survival and (A) classification of treatment beyond progression (TBP) drugs. (B) treatment of the non-TBP group. (C) region. [file DataSheet_1.zip › Supplemental Figure 3C.tif]
