# Supplementary material for: Treatment beyond progression in non-small cell lung cancer: A systematic review and meta-analysis
Source: Front Oncol. 2022 Nov 17;12:1023894. doi: 10.3389/fonc.2022.1023894 (PMC9713814; doi:10.3389/fonc.2022.1023894)
Supplement: Supplementary file 2 [file Table_1.docx]

Supplemental table 1. A summary of risk of bias assessment using the Risk of Bias Assessment Tool for Non-randomized Studies (RoBANS)

|  | Selection | | Performance | Detection | Attrition | reporting |
| --- | --- | --- | --- | --- | --- | --- |
|  | Selection of participants | Confounding variables | Measurement of exposure | Blinding of outcome assessments | Incomplete outcome data | Selective outcome reporting |
| Faehling et al, 2013 | high | unclear risk | low | low | low | low |
| Nishino et al, 2013 | low | high | low | unclear risk | low | low |
| Ou et al, 2014 | low | low | low | low | low | low |
| Chiari et al, 2015 | low | low | low | unclear risk | low | low |
| Auliac et al, 2016 | low | low | low | low | low | low |
| Higashiguchi et al, 2016 | low | high | low | high | low | low |
| Leon et al, 2016 | low | low | low | high | low | low |
| Moiseyenko et al, 2016 | high | low | low | unclear risk | low | low |
| Song et al, 2016 | low | high | low | unclear risk | low | low |
| WANG et al, 2016 | low | low | low | low | low | low |
| Ding et al, 2017 | low | low | low | unclear risk | low | low |
| Le et al, 2018 | low | high | low | high | high | low |
| Mehlman et al, 2019 | low | high | low | high | low | low |
| Metro et al, 2019 | low | low | low | high | low | low |
| Mu et al, 2019 | low | high | low | low | low | low |
| Ricciuti et al, 2019 | low | low | low | low | low | low |
| Xing et al, 2019 | low | high | low | high | low | low |
| Zhao et al, 2019 | low | low | low | unclear risk | low | low |
| Cortellini et al, 2020 | low | low | low | high | low | low |
| Ge et al, 2020 | low | low | low | unclear risk | low | low |
| Liang et al, 2020 | low | high | low | low | low | low |
| Stinchcombe et al, 2020 | low | low | low | high | low | low |
| Won et al, 2020 | low | low | low | low | low | low |
| Enomoto et al, 2021 | low | low | low | high | low | low |
| Heo et al, 2021 | low | high | low | low | low | low |
| Xu et al, 2021 | low | high | low | unclear risk | low | low |
| Gandara et al, 2018 | low | low | low | low | low | low |
