# Supplementary material for: Treatment beyond progression in non-small cell lung cancer: A systematic review and meta-analysis
Source: Front Oncol. 2022 Nov 17;12:1023894. doi: 10.3389/fonc.2022.1023894 (PMC9713814; doi:10.3389/fonc.2022.1023894)
Supplement: Supplementary file 3 [file Table_2.docx]

Supplemental Table 2. A summary of risk of bias assessment using the Revised Cochrane risk-of-bias tool for randomized trials (RoB 2)

|  | randomization process | intended intervention | missing outcome data | measurement of outcome | selection of reported result | overall |
| --- | --- | --- | --- | --- | --- | --- |
| HALMOS et al, 2015 | some | high | high | some | some | high |
| Takeda et al, 2016 | low | high | low | low | some | high |
| Mok et al, 2017 | low | low | low | low | low | low |
| Gridelli et al, 2018 | some | low | low | low | low | some |
| Schuler et al, 2016 | some | low | low | low | low | some |
